# Application of NPE in the assessment of a patent ductus arteriosus

**DOI:** 10.1038/s41390-018-0077-x

**Published:** 2018-08-02

**Authors:** David van Laere, Bart van Overmeire, Samir Gupta, Afif El Khuffash, Marilena Savoia, Patrick J McNamara, Christoph E Schwarz, Willem P de Boode, on behalf of the European Special Interest Group “Neonatologist Performed Echocardiography” (NPE), WP de Boode, T Austin, K Bohlin, MC Bravo, CR Breatnach, M Breindahl, E Dempsey, A El-Khuffash, AM Groves, S Gupta, B Horsberg Eriksen, PT Levy, PJ McNamara, Z Molnar, E Nestaas, SR Rogerson, CC Roehr, M Savoia, U Schubert, CE Schwarz, A Sehgal, Y Singh, MG Slieker, C Tissot, R van der Lee, D van Laere, B van Overmeire, L van Wyk

**Affiliations:** 1 Department of Neonatal Intensive Care, University Hospital Antwerp, Edegem, Belgium; 2 Department of Neonatology, Hôpital Erasme, Université Libre de Bruxelles, Brussels, Belgium; 3 Neonatal Medicine, University Hospital of North Tees, Durham University, Stockton-on-Tees, United Kingdom; 4 Department of Neonatology, The Rotunda Hospital, Dublin, Ireland; 5 Department of Paediatrics, The Royal College of Surgeons in Ireland, Dublin, Ireland; 6 Neonatal Intensive Care Unit, Azienda Sanitaria Universitaria Integrata, Udine, Italy; 7 Departments of Pediatrics and Physiology, University of Toronto, Toronto, Canada; 8 Department of Neonatology, University Children’s Hospital, Tuebingen, Germany; 9 Department of Neonatology, Radboud university medical center, Radboud Institute for Health Sciences, Amalia Children’s Hospital, Nijmegen, The Netherlands; 10 Department of Neonatology, Rosie Hospital, Cambridge University Hospitals NHS Foundation Trust, Cambridge, United Kingdom; 11 Department of Neonatology, Karolinska University Hospital, Karolinska Institutet, Stockholm, Sweden; 12 Department of Neonatology, La Paz University Hospital, Madrid, Spain; 13 Karolinska University Hospital, Karolinska Institutet, Stockholm, Sweden; 14 INFANT Centre, Cork University Maternity Hospital, University College Cork, Cork, Ireland; 15 Department of Pediatrics, The Royal College of Surgeons in Ireland, Dublin, Ireland; 16 Division of Newborn Medicine, Mount Sinai Kravis Children’s Hospital, New York, NY USA; 17 University Hospital of North Tees, Durham University, Stockton-on-Tees, United Kingdom; 18 Department of Pediatrics, Møre and Romsdal Hospital Trust, Ålesund, Norway; 19 Department of Pediatrics, Washington University School of Medicine, Saint Louis, MO USA; 20 Department of Pediatrics, Goryeb Children’s Hospital, Morristown, NJ USA; 21 John Radcliffe Hospital, Oxford, United Kingdom; 22 Institute of Clinical Medicine, Faculty of Medicine, University of Oslo, Oslo, Norway; 23 Department of Cardiology and Center for Cardiological Innovation, Oslo University Hospital, Rikshospitalet, Oslo, Norway; 24 Department of Paediatrics, Vestfold Hospital Trust, Tønsberg, Norway; 25 The Royal Women’s Hospital, Parkville, VIC Australia; 26 Department of Paediatrics, University of Oxford, John Radcliffe Hospital, Oxford, United Kingdom; 27 Azienda Ospedaliero-Universitaria S Maria della Misericordia, Udine, Italy; 28 Department of Clinical Science, Intervention and Technology, Karolinska Institutet, Stockholm, Sweden; 29 Department of Neonatology, University Children’s Hospital of Tübingen, Tübingen, Germany; 30 Department of Pediatrics, Monash University, Melbourne, Australia; 31 Addenbrooke’s Hospital, Cambridge University Hospitals NHS Foundation Trust, Cambridge, United Kingdom; 32 Department of Paediatric Cardiology, Radboudumc Amalia Children’s Hospital, Nijmegen, The Netherlands; 33 Department of Pediatrics, Clinique des Grangettes, Chêne Bougeries, Switzerland; 34 Department of Pediatrics, Antwerp University Hospital UZA, Edegem, Belgium; 35 Department of Neonatology, University Hospital Brussels, Brussels, Belgium; 36 Department of Paediatrics & Child Health, University of Stellenbosch, Cape Town, South Africa

## Abstract

In many preterm infants, the ductus arteriosus remains patent beyond the first few days of life. This prolonged patency is associated with numerous adverse outcomes, but the extent to which these adverse outcomes are attributable to the hemodynamic consequences of ductal patency, if at all, has not been established. Different treatment strategies have failed to improve short-term outcomes, with a paucity of data on the correct diagnostic and pathophysiological assessment of the patent ductus arteriosus (PDA) in association with long-term outcomes. Echocardiography is the selected method of choice for detecting a PDA, assessing the impact on the preterm circulation and monitoring treatment response. PDA in a preterm infant can result in pulmonary overcirculation and systemic hypoperfusion, Therefore, echocardiographic assessment should include evaluation of PDA characteristics, indices of pulmonary overcirculation with left heart loading conditions, and indices of systemic hypoperfusion. In this review, we provide an evidence-based overview of the current and emerging ultrasound measurements available to identify and monitor a PDA in the preterm infant. We offer indications and limitations for using Neonatologist Performed Echocardiography to optimize the management of a neonate with a PDA.

## Introduction

The significance of a patent ductus arteriosus (PDA) and its impact on long-term cardiorespiratory health remain areas of ongoing debate in neonatology.^1,2^ Even the persistent patency of a PDA is a clinical conundrum, as most premature neonates in whom the PDA fails to close in the first week of age or even by the time of discharge will undergo spontaneous closure without experiencing significant cardiac morbidity.^3,4^ Although prolonged patency may be associated with numerous adverse outcomes, the extent to which these adverse outcomes are attributable to the hemodynamic consequences of ductal patency, if at all, has not been established.^5^ Currently, echocardiography is the most clinically applicable modality for identifying a PDA, with Neonatologist Performed Echocardiography (NPE) potentially playing an essential role in devising treatment options and assessing their impact on the preterm circulation. Centers that develop NPE may greatly benefit from the “unfettered” access to echocardiography with a targeted approach that focuses on the high-risk patient population with specific hemodynamic characteristics receiving treatment.

The purpose of this review is not to advise on treatment options, but to provide a practice guideline on how to evaluate hemodynamic significance and characterize shunt volume using NPE. Specifically, in this review, we discuss the current and emerging ultrasound measurements available to identify and monitor a PDA in the preterm infant. We offer recommendations for using NPE to optimize the management of a neonate with a PDA and understand its contribution to overall pathophysiology in neonates.

### Epidemiology, symptomatology, and pathophysiology of the PDA

The ductus arteriosus and foramen ovale are essential components of the fetal circulation. During pregnancy, these shunts divert oxygenated blood from the placenta away from the pulmonary circulation towards the systemic circulation (RtL = right-to-left shunt). A normal postnatal transition is characterized by a fall in pulmonary vascular resistance, accompanied by a rise in systemic vascular resistance. These physiological changes lead to a reversal of the shunt through the PDA away from the systemic circulation toward the pulmonary circulation (LtR = left-to-right shunt). In the majority of term infants, the postnatal transition ends with functional closure of the PDA in the first 72 postnatal hours after a process of ductal constriction followed by anatomical remodeling.^6–8^

In preterm infants, the ductus arteriosus is at increased risk of patency beyond the first days of life. This is related to the histological immaturity of the PDA. With advancing development in utero, there is progressive intimal thickening with formation of intimal cushions making the PDA more susceptible for degeneration and remodeling.^9,10^ Important risk factors for persistent ongoing patency of the ductus arteriosus are lower gestational age, lack of antenatal steroids, low platelet count in the first days of life, and need for mechanical ventilation.^11–13^

**Fig. 1:**
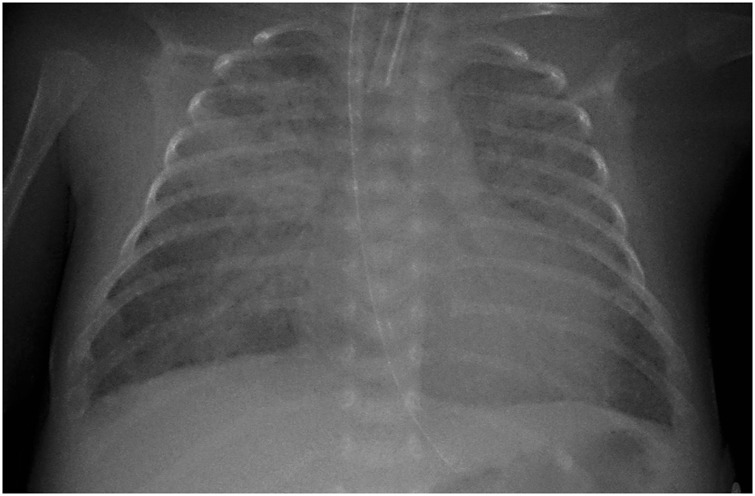
Chest radiograph in a ventilated preterm infant with a large PDA. Note the large heart shadow and the increased lung markings representing pulmonary overcirculation

The incidence of a PDA in babies born <1500 g varies between 18 to 77%.^14,15^ However, delayed spontaneous closure of the PDA occurs frequently in these patients. In patients with a birth weight >1000 g, a spontaneous closure rate of 94% before discharge has been reported.^16^ In a large prospective study of extreme low birth weight (ELBW) infants with serial daily echocardiography, spontaneous permanent closure of the PDA occurred in 34% of the population by day 8 of life.^17^ Recent retrospective cohort studies of extreme low gestational age infants have reported a spontaneous closure rate of 73% in surviving infants at 2 months of postnatal age^18^ up to 95% at an average of 44 days.^19^ The effect of the PDA on the preterm circulation is caused by the volume of the shunt through the duct. Ductal flow is modulated mainly by the pressures at both ends of the shunt. In the early hours of life, relatively high pulmonary pressures result in a balanced pulmonary to systemic circulation. Signs of a shunt effect (diastolic hypotension, worsening ventilation, pulmonary hemorrhage) develop when the shunt volume increases secondary to an increased pressure gradient (Fig. 1). A heart murmur, hyperactive precordium, bounding pulses, and a widened systolic to diastolic pulse pressure amplitude are recognized clinical signs attributed to the presence of a hemodynamically significant PDA (HsPDA). Most clinical signs lack sensitivity in the first days of life and hence during this period, a PDA is usually diagnosed by echocardiography.^20–22^

The presence of a PDA in a preterm infant can result in pulmonary overcirculation and systemic hypoperfusion. The PDA is associated with several morbidities including intraventricular hemorrhage (IVH), necrotizing enterocolitis (NEC), bronchopulmonary dysplasia (BPD), and death.^23–26^ Different treatment strategies have been studied over the years. These include prophylactic treatment, early targeted treatment, treatment of a clinically symptomatic PDA, and the conservative approach of “watchful waiting.” Although some trials of pharmacological therapy have shown a reduction in short-term morbidities such as pulmonary hemorrhage and severe IVH,^27–30^ data on long-term outcomes are limited to one large trial of prophylactic treatment using indomethacin showing no beneficial effect in the treatment group.^30^ Currently, opinion is divided regarding “if,” “when,” and “how” to treat a PDA.^31^ It needs to be acknowledged that there is marked heterogeneity of the definition of a HsPDA with limited consideration of the magnitude of the shunt volume; in some cases, the entry criterion for trials of therapeutic intervention is based on mere patency of the ductus arteriosus.^32,33^ The possible co-linearity between the presence of a PDA, lower gestational age, and most of the outcomes measured, make it difficult to accept a “one size fits all” approach to the PDA. Rather, the PDA should be considered as a dynamic physiologic shunt that at a certain threshold or after a specific duration of exposure, may become harmful.

## Application of NPE in the evaluation of PDA

A significant shunt through the PDA that remains clinically “silent” may occur in the first days of life. The development of echocardiographic signs of hemodynamic significance precedes the development of clinical signs by a mean of 2 days.^21^ Echocardiography is currently the preferred tool for predicting or diagnosing ductal patency and assessing hemodynamic significance.^34^ There is some evidence that serial echocardiography facilitates early identification of a HsPDA and might reduce morbidity associated with the PDA.^35,36^

There are important considerations when using echocardiography which were recently set out by the European consensus statement on NPE.^37^ Firstly, it is important that measures are taken to ensure the patients’ comfort. Secondly, the initial scan should be a comprehensive appraisal of cardiac anatomy, sufficient to confirm structural normality of the heart and avoid inadvertent PDA treatment in the presence of a duct-dependent lesion. Finally, the clinician must be aware that echocardiographic indices of hemodynamic significance have variable reproducibility between observers.^38,39^ It is important to strive for consistency between operators on where and how to measure the different variables involved in the assessment of the PDA.

### Assessment of hemodynamic significance of the PDA

The traditional view of the PDA as a dichotomous entity based on transductal dimension represents a physiologic oversimplification. Assessment of transductal diameter alone is confounded by image quality, vessel geometry, and the dynamic nature of the vessel. Although direct calculation of the shunt volume is not feasible, clinicians can assess surrogate measures of pulmonary overcirculation and/or systemic steal phenomena. The concordance between PDA diameter and indices of shunt volume is weak, which might be related to the effect of transductal pressure gradient being a more important determinant of shunt volume.^39^ A standard definition of a HsPDA is lacking, and therefore, the validity of those surrogate markers is unproven. Some authors have used alternative echocardiographic measurements of ductal flow^40,41^ or assessed ductal flow by using a different imaging technique.^42^ A PDA staging system that also incorporates clinical conditions has been suggested.^43^ A recent retrospective study, using the echocardiographic staging system, showed that patients with prolonged exposure to a large PDA had an increased risk of BPD.^26^

**Fig. 2:**
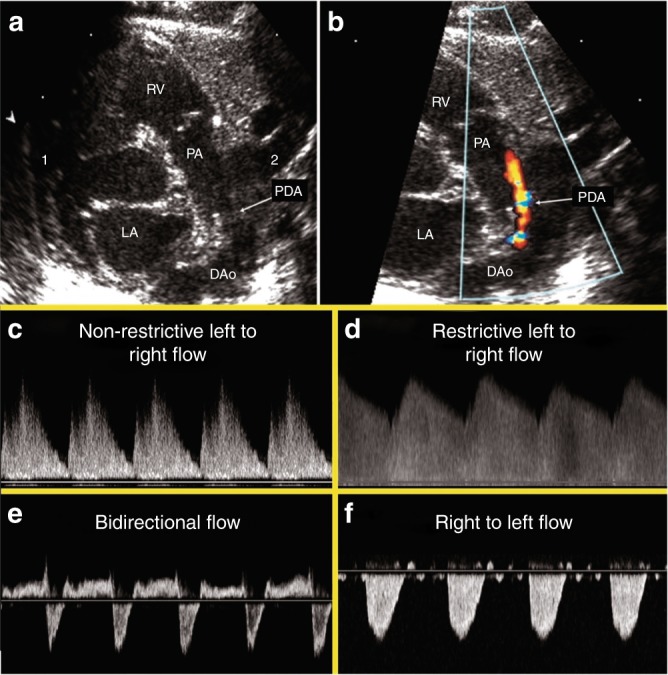
PDA 2D, color Doppler image and Doppler flow patterns. The top panels demonstrate the PDA in 2D (**a**) and color Doppler (**b**). **c** Pulsatile or non-restrictive pattern: characterized by a left to right (LtR) shunt with an arterial waveform and high peak systolic velocity: end-diastolic velocity ratio. **d** Restrictive pattern: characterized by high systolic and diastolic velocity, and low peak systolic velocity: end-diastolic velocity ratio. **e** Bidirectional pattern: elevated pulmonary pressures equal to or near systemic. **f** Right to left (RtL) flow: supra-systemic pulmonary pressures

**Fig. 3:**
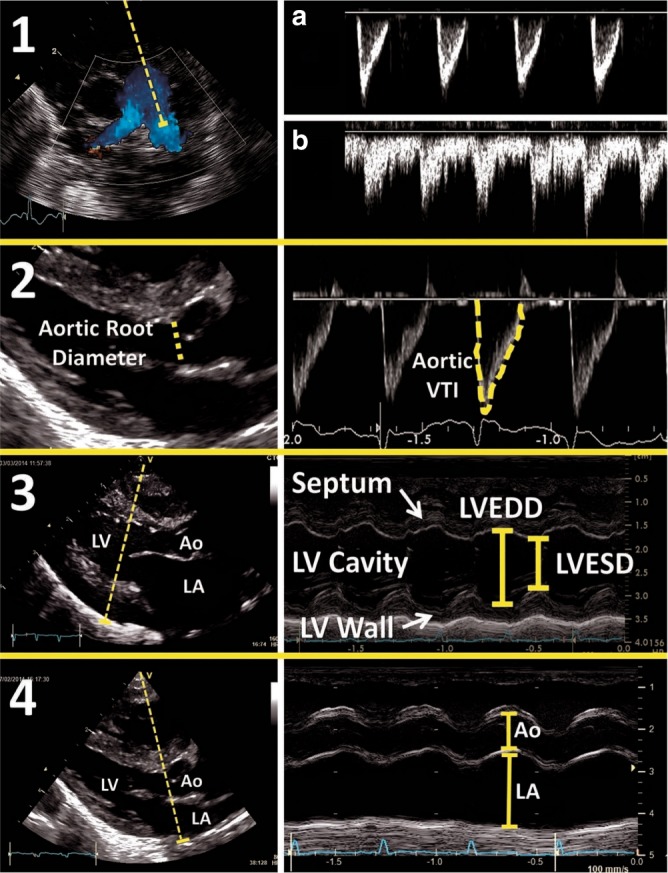
Assessment of left heart volume loading. (1) Measurement of diastolic flow in the left pulmonary artery. **a** Normal situation without ductal left-to-right shunting. **b** Illustrates forward diastolic flow in the presence of significant left-to-right ductal flow. (2) Measurement of LVO: increased LVO in the setting of a PDA indicated increased pulmonary venous return. (3) Measurement of LV diameter in diastole: increased LV diameter is another surrogate marker for increased LV end-diastolic volume. (4) LA:Ao ratio: atrial enlargement can be indexed to a relatively fixed aortic root diameter to further estimate in degree of increased LA volume

**Fig. 4:**
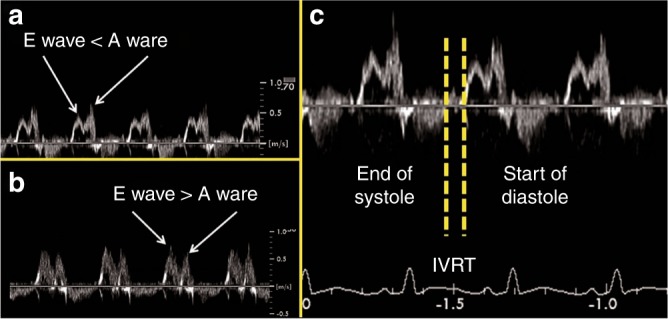
Assessment of left heart pressure loading. Transmitral LV filling in normal term infants is characterized by a predominance of early diastolic (“E”) filling, with limited late LV filling occurring during atrial contraction (“A”), resulting in an E:A ratio >1. **a** Healthy preterm infants without a PDA have intrinsically decreased LV diastolic function, relying more on late atrial filling, and E:A ratio <1. **b** and **c** Preterm infants with a large PDA have increased left atrial pressure which results in earlier mitral valve opening and drives early passive filling, resulting in shortened isovolumic relaxation time (<40 ms) and a “pseudonormalized” E:A ratio >1

**Fig. 5:**
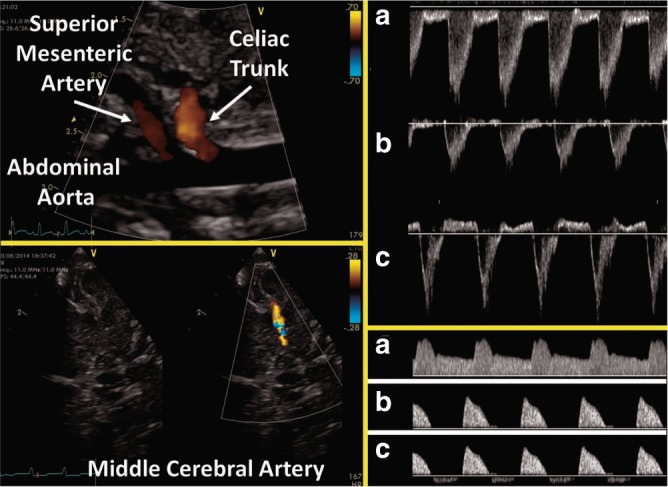
Assessment of diastolic flow in a post-ductal artery. Measurement of pulsed wave Doppler pattern in the celiac trunk, the abdominal aorta, and the middle cerebral can highlight the effect of left-to-right shunting across the PDA. In the top Doppler panel, three abdominal aortic Doppler wave forms are illustrated demonstrating normal forward diastolic flow (**a**), absent diastolic flow (**b**), and revered diastolic flow (**c**). A similar pattern can be seen in the lower Doppler panel which is representative of celiac and middle cerebral arteries

In an attempt to standardize the echocardiographic assessment of hemodynamic significance of a PDA, we propose that a comprehensive NPE study of the PDA should include information on (a) PDA characteristics—diameter, flow direction, (ratio of) systolic and diastolic flow velocities (Fig. 2); (b) Indices of pulmonary overcirculation—left ventricular output + one parameter of left-sided volume loading (Fig. 3) OR left heart pressure loading (Fig. 4); (c) Systemic shunt effect—Doppler flow patterns in the systemic circulation (Fig. 5).

Below is a detailed description on how to image and interpret the different echocardiographic variables related to the assessment of the PDA. The essential echocardiographic requirements for the assessment of hemodynamic significance of a PDA are summarized in Table 1.

**Table 1:** Essential echocardiographic requirements for the assessment of hemodynamic significance of a PDA

1) PDA characteristics of dimension and flow
a) diameter (mm)
b) flow direction (left to right, bidirectional with right to left ≤ or >30% of the cardiac cycle, right to left)
c) velocity in systole and diastole (m/s) and gradient
2) Indices of pulmonary overcirculation
a) LVO (mL/kg/min)
b) left heart volume loading: choose one parameter:
- La:Ao, LVEDD (mm)
- Pulmonary vein d wave velocity (m/s)
- LPA diastolic velocity (m/s)
c) left side pressure loading: choose one parameter:
- Mitral valve E:A - IVRT (ms)
3) Indices of systemic shunt effect
a) Flow direction in one of the following post-ductal artery
- aorta descendant or
- celiac trunk
- middle cerebral artery (forward, absent, reversed)

### PDA characteristics: patency, dimension, and direction of the shunt

Although PDA can be visualized from many windows, the high left-sided parasternal “ductal” view is the preferred window to obtain a clear 2D image of the ductus arteriosus. The ultrasound probe is placed in a true sagittal plane to the left of the sternum with the marker pointing toward the head to obtain the ductal view. The PDA is visualized as a structure leaving the left side of the junction of the main pulmonary artery and the left pulmonary artery (LPA) toward the descending aorta. Color Doppler may be used to visualize the direction of transductal blood flow. A LtR shunt is easily seen as the jet is red in color; however, a RtL shunt may be missed as the blue jet can be mistaken for pulmonary artery or aortic arch flow.

Dimension of the duct is approximated by the diameter of the PDA. When the duct is large, the diameter can be obtained on a plain 2D image but there is often a variable degree of constriction. This is sometimes more apparent on color Doppler imaging. Variability in color gain setting can produce variability in measurements. Best practice is to increase the gain until background noise appears outside the vessels, then reducing it back until the noise is suppressed. It has been proposed to measure the PDA at the smallest dimension (site of maximum constriction) by a frame by frame analysis, which is usually at the pulmonary end.^44^ The diameter can be expressed as an absolute value (smallest cut-off 1.5 mm) or indexed either to the dimension of the LPA (smallest cut-off 0.5)^5^ or to the patient’s weight (cut-off 1.4 mm/kg).^40^ Transductal diameter has been an important marker used in many clinical studies, though there is some evidence of significant variability between observers, with a 95% limit of agreement between two observers of 48% for PDA diameter.^45^

Velocity, direction, and volume of the shunt are determined by the dimension of the shunt and the relative pressures at both ends. As such, a similar size duct can have a varying shunt volume depending on the gradient between the pulmonary and systemic pressure. Velocity and direction of the shunt during the cardiac cycle can be obtained by applying a pulsed or continuous wave Doppler signal in the PDA. The direction of the shunt is either LtR, bidirectional with RtL shunting ≤30% of the cardiac cycle, bidirectional with RtL shunting >30% of the cardiac cycle, or a pure RtL. The last two patterns may be pathological and should trigger the examiner to consider duct-dependent cardiac lesions and/or elevated pulmonary pressures, both of which are contra-indications to medical ductal closure. It is important to appreciate that there may be some babies with a chronic high volume shunt through the PDA that present with bidirectional but predominantly left to right flow, who may benefit from treatment.

Non-restrictive shunts have a low peak systolic velocity with a high systolic to diastolic velocity gradient (Vmax:Vmin). Restrictive shunts are in general characterized by a high peak systolic velocity and a low systolic to diastolic velocity gradient. A retrospective study by Condo et al. showed that flow patterns and ductal diameter during early life were significantly associated.^46^ Based on the flow pattern and velocities, the PDA can be grouped into closing (Vmax: Vmin <2.0), pulsatile (Vmax: Vmin ≥2.0), growing (right to left <30% of cycle), and right-to-left (right to left >30% of cycle time) pattern (Fig. 2).^47^ It is important to emphasize that these studies were performed during the first days of life. At a later age, chronic high-volume shunts can present with high peak systolic velocity and may be incorrectly labeled as restrictive.

### Echocardiographic parameters associated with pulmonary overcirculation

#### Left heart volume loading

In presence of neonatal shunts, the left ventricular output (LVO) represents the systemic blood flow plus the shunt across the PDA. It can be measured according to the method described by Kluckow and Evans.^48^ A large left-to-right shunt through the PDA leads to an increase in LVO. A calculated output of more than 300 mls/kg/min was reported to be associated with the clinical presence of a PDA.^49^ There are marked changes in cardiac output during the perinatal transition making this parameter difficult to use for the assessment of the PDA during the first days of life. One should be cautious when the LVO is low or normal in the presence of a large PDA as this may be influenced by a concomitant trans-atrial shunt or may be lower than expected due to a failing myocardium unable to compensate for increased demand. A ratio of LVO and superior vena cava (SVC) flow (representing upper body blood flow) (LVO/SVC ratio) has been suggested as a marker for severity of ductal flow. In a prospective study of preterm infants below 30 weeks of gestation, the mean LVO/SVC ratio was 2.3 in patients with a closed duct. The authors chose a LVO/SVC ratio ≥4 to define a HsPDA.^40^ “However, in this study is was concluded that assessment of LVO/SVC ratio does not provide additional benefit to the overall assessment of PDA significance. The more conventional markers including LA/Ao ratio, ductal diameter, mean and end-diastolic flow velocity in the LPA are more accurate and easier to measure.”

Presence of forward pulmonary flow in diastole in the LPA is indicative of a significant LtR shunt through the PDA. The LPA can be visualized from a high parasternal short axis view using color Doppler. Mean and end-diastolic velocity is quantified by using pulsed wave Doppler in the LPA and tracing the Doppler signal. Both parameters correlated well with a high transductal flow, with cut-off points of 0.42 m/s and 0.20 m/s, respectively.^40^ High pulmonary blood flow secondary to a significant LtR shunt leads to increased pulmonary venous return and a higher LA preload. Left atrium to aortic root ratio (LA:Ao), left ventricular end-diastolic diameter (LVEDD), and pulmonary vein diastolic wave velocity can be used as surrogate markers for pulmonary venous return.

LA:Ao and LVEDD may be measured from the parasternal long axis view using M-mode with the cursor perpendicular to the aorta at the level of the aortic valve or to the septum at the tip of the mitral valve leaflets, respectively. LA:Ao is frequently used in many clinical trials. The most commonly used cut-off value for LA:Ao is 1.5.^43,50^ Normal reference ranges for left ventricular dimensions in preterm infants in relation to body weight and postnatal age have been published.^51,52^ Using *z*-scores for LVEDD, the effect of volume loading on the left ventricle can be evaluated over time. When assessing volume loading of the left heart, it is important to understand that preload is not only dependent on the PDA and it is advisable to evaluate the presence of inter-atrial shunting. A large left-to-right shunt through the foramen ovale may “offload” the left side of the heart even in the presence of a significant shunt through the PDA leading to an artificially low/normal LA:Ao ratio. This measurement is also prone to a high degree of inter-observer variability.

In a high parasternal long axis view, with the probe slightly rotated clockwise, the pulmonary veins can be visualized entering the left atrium (“crab view”) using color Doppler imaging with the scale lowered to 30–40 cm/s. By applying a pulsed wave Doppler signal in the lower pulmonary veins (to minimize level of insonation) one can obtain a flow pattern with a systolic (S-wave), diastolic (D-wave), and atrial contraction (A-wave) component. Although there is limited data at present to support this new variable, a peak velocity cut-off of 0.5 m/s for the D-wave may be indicative of a moderate shunt.^39^ Figure 3 gives on overview of the echocardiographic assessment of left heart volume loading.

#### Left heart pressure loading

The mitral valve E to A wave ratio (E/A ratio) can be obtained from a four-chamber view with the probe positioned on the apex of the heart with the marker facing the left side. The probe must be tilted to the right shoulder and the pulsed Doppler range gate should be set slightly below the mitral valve annulus. Mitral valve E/A ratio refers to the ratio of the velocity of the early (E) diastolic phase of ventricular filling versus the late atrial (A) contraction component. In preterm infants, due to immaturity of the myocardium, there is moderate impairment of diastolic performance leaving the ratio usually <1. In the presence of a significant PDA, atrial pressure increases which leads to a reversal of the E/A ratio >1. Isovolumic relaxation time (IVRT) is the time between the closure of the aortic valve and the opening of the mitral valve. IVRT decreases in the presence of a significant PDA. This is related to an earlier opening of the mitral valve in the presence of higher left atrial pressure. IVRT can either be calculated from the Doppler image of mitral valve E/A ratio or obtained with Tissue Doppler Imaging (TDI) interrogating the left ventricular posterior wall in an apical four-chamber view. In a serial Doppler study of very low birth weight infants, IVRT was lower in patients with PDA compared to those with a closed duct (mean 45±7 ms vs. 55±5 ms).^53^ Figure 4 gives an overview of the echocardiographic assessment of left heart pressure loading.

### Echocardiographic assessment of systemic shunt effect

Although the PDA shunts blood away from the systemic circulation throughout the cardiac cycle, this becomes more apparent during diastole. Doppler flow patterns from the descending aorta can be obtained from a high parasternal view with the Doppler sample gate set distal to the PDA. In a sagittal abdominal view with the marker pointing toward the head, the celiac trunk and mesenteric artery can be identified by lowering the color Doppler velocity range. Using pulsed wave Doppler, three patterns of diastolic flow can be distinguished: forward, absent, and retrograde flow. A study using magnetic resonance imaging (MRI) quantified the volume of the ductal shunt as a percentage of LVO based on the calculated difference between LVO and total systemic blood flow. Reversed diastolic flow in the post-ductal aorta on echocardiography was the best predictor of MRI-derived high volume ductal shunt.^42^

To assess a potential steal phenomenon on the cerebral blood flow in the presence of a PDA, flow direction and velocity in cerebral arteries are frequently assessed using cranial ultrasound. The middle cerebral artery should be visualized from a temporal window using color Doppler imaging to minimize angle of insonation. A retrograde or absent flow pattern on Pulsed wave Doppler in diastole is indicative of a large shunt. Resistive index (RI = (peak systolic velocity−end-diastolic velocity) / peak systolic velocity) can be calculated and is often used as a surrogate parameter for cerebral blood flow. In a recent large prospective study interrogating multiple cerebral arteries by Doppler ultrasound, a significant elevated RI in the anterior cerebral artery and right internal carotid artery was seen in patients with a HsPDA compared to neonates with a closed duct.^54^ To date the clinical relevance of Doppler flow patterns remain unknown.^55^ Figure 5 gives an overview of the echocardiographic assessment of diastolic flow in a post-ductal artery.

### Reproducibility of echocardiographic measures

The literature on the feasibility and reproducibility of most ultrasound variables is summarized in Table 2 with information on repeatability, echocardiographic views, limitations, and cut-off values in relation to different categories of significance of a PDA.

**Table 2:** Echocardiography parameters of ductal hemodynamic significance

Parameter	Variable	Repeatability	Echo view	Echo Mode	Limitations	Cut-off value		
						Small shunt	Moderate shunt	Large shunt
*Morphology of the PDA*	PDA diameter (mm)		Ductal view	2D, color Doppler		<1.5	1.5–2.0	>2.0
	PDA:LPA ratio		Ductal view high parasternal view	Calculated	Within first 96 h	<0.5	0.5–1	>1
	PDA diameter to body weight	0.85 (0.68–0.94)^a^; 21 (12–112)^c^	Ductal view	2D, color Doppler calculated				≥1.4 mm/kg
*Doppler of the PDA*	PDA vmax (cm/s)		Ductal view: pulmonary end	PWD or CWD	Within first 72 h	>2	1.5–2.0	<1.5
	Ratio systolic to diastolic velocity ratio		Ductal view: pulmonary end	PWD or CWD	Caveat chronic high volume shunts	<2	2–4	>4
*Pulmonary overcirculation*	LA:Ao	0.65 (0.44–0.82)^a^; 16 (12–23)^c^	Parasternal long axis view	M-mode	Influenced by atrial shunt	<1.5	1.5–2.0	>2.0
	LVEDD	0.93 (0.86–0.97)^a^	Parasternal long axis view	M-mode	Influenced by atrial shunt			
	LVO (ml/kg/min)	0.97 (0.94–0.99)^a^	Parasternal long axis view + apical five-chamber view	PWD calculated	Limited use during transition	<200	200–300	>300
	End-diastolic LPA flow velocity (m/s)		High parasternal view	PWD		<0.2	0.2–0.5	>0.5
	IVRT (ms)	0.84 (0.63–0.93)^a^	Subcostal four -chamber view	PWD / TDI		>40	30–40	<30
	Pulmonary vein d wave velocity (m/s)		High suprasternal view	PWD		<0.3	0.3–0.5	>0.5
	Mitral valve E/A ratio	0.9 (0.77–0.95)^a^	Subcostal four -chamber view	PWD		<1	1	>1
*Systemic hypoperfusion*	DADF	0.75 (0.43–1.00)^b^	High parasternal	PWD		Forward	Absent	Reversed
	MCA		Cranial cross-sectional	PWD		Forward	Forward	Absent/reversed
	CAF	0.88 (0.65–1.00)^b^	Abdominal saggital	PWD		Forward	Absent	Reversed

*Ao* aortic root, *CAF* celiac artery diastolic flow, *CWD* continuous wave Doppler, *DADF* descending aorta diastolic flow, *IVRT* isovolumic relaxation time, *LA* left atrium, *LPA* left pulmonary artery, *LVEDD* left ventricular end-diastolic diameter, *LVO* left ventricular output, *PWD* pulsed wave Doppler, *SVC* superior vena cava, *TDI* tissue Doppler imaging

^a^ Lin’s concordance coefficient (95% CI)

^b^ Kappa coefficient (95% CI)

^c^ Repeatability index (95% CI)

## Refining the use of NPE in the management of the PDA

### Early prediction of a symptomatic PDA

**Fig. 6:**
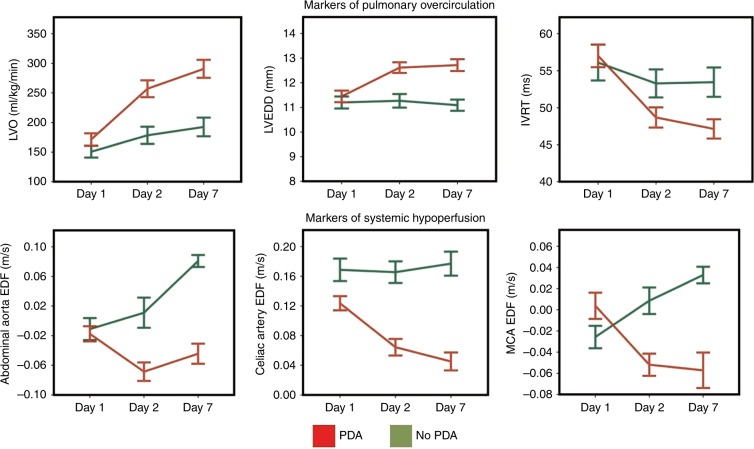
Patterns of echocardiography markers in infants with and without a PDA over the first week of age. Divergence in echocardiography parameters becomes apparent within the first 48 h following birth. Data represent means and standard error (data adapted from EL-Khuffash et al.^62^). LVO left ventricular output, LVEDD left ventricular end-diastolic diameter, IVRT isovolumic relaxation time, EDF end-diastolic velocity, MCA middle cerebral artery

Identifying early echocardiographic predictors of ductal patency might lead to a more targeted approach. This approach could potentially reduce side-effects of the current therapy by avoiding unnecessary treatment and reduce prolonged exposure to a potential harmful shunt. Several studies have addressed this issue and identified different variables with varying predictive capacity (see Tables 2 and 3).^5,47,56–61^ Most parameters have good specificity but lack sensitivity. The studies differed significantly by the timing of the initial echocardiography, study population, and endpoint. A ductal diameter ≥1.5 mm within the first 31 h, a first appearing Doppler pulsatile ductal flow pattern, and a ratio of systolic to end-diastolic ductal flow velocity >1.9 within the first 48 h are the predictors with highest sensitivity and specificity for patency. Recently, a PDA severity score based on echocardiographic variables of pulmonary overcirculation and diastolic LV function was shown to be significantly associated with chronic lung disease or death.^62^ During this study, serial echocardiography showed marked divergence in echocardiographic parameters between infants with and without PDA (in the first week of life (Fig. 6)). Currently, one prospective trial studied a targeted approach based on echocardiographic prediction of patency. Unfortunately, the study remained underpowered to test the primary outcome, but there was a significant reduction in pulmonary hemorrhage in the treatment group.^27^ A recent national based cohort study showed that an early echocardiographic screening of the PDA was associated with lower in-hospital mortality and likelihood of pulmonary hemorrhage.^36^

**Table 3:** Early predictive echocardiographic variables for the development of a hemodynamic significant PDA

Author	GA/BW included	Timing of first echo	Endpoint	Parameters	Sensitivity (%	Specificity (%)
Kluckow and Evans, 1995^46^	<1500 g ventilated	<31 h	Diagnosis of a significant PDA meeting clinical and echocardiographic criteria (1–15 d)	PDA diameter ≥1.5 mm	81	85
				DADF absent/retrograde	68	85
				LA:Ao ≥1.5	29	91
				LVO ≥300 ml/kg/min	26	92
Su et al. ^47^	<1500 g ventilated	Daily echo for 7 days	HsPDA > 2 clinical, radiological signs and echocardiographic signs of L–R shunt	First growing pattern	64	81
				First pulsatile pattern	93	100
Kwinta et al.^49^	GA 24–32 weeks	12–48 h after birth	Significant PDA requiring surgical ligation	PDA diameter >1.5 mm/kg	94	73
				fE >36 cm/s	70	62
				CI Ao >2.5 L/min/m^2^	82	64
				PDA diameter >1.5 mm/kg + FiO2 >0.3	81	84
Ramos et al.^36^	BW < 1000 g	Echo before 4 days of age	Need for subsequent treatment based on clinical and echocardiographic signs	PDA:LPA ≥0.5	78	80
Harling et al.^45^	GA < 32 weeks	Echo at 24 h of age	Need for subsequent treatment based on clinical and echocardiographic signs	PDA diameter ≥2 mm/kg	91	59
				Pulsatile flow pattern	91	59
				LA:Ao ≥1.4	70	29
				Green pixel on color Doppler	64	47
Harling et al.^45^	GA < 32 weeks	Echo at 72 h of age	Need for subsequent treatment based on clinical and echocardiographic signs	PDA diameter ≥2 mm/kg	89	70
				Pulsatile flow pattern	67	78
				LA:Ao ≥1.4	56	50
				Green pixel on color Doppler	70	44
Thakavel et al.^48^	GA ≤ 30 weeks	Echo at 3 days of life	Spontaneous PDA closure on late echocardiography without treatment	*For GA* *≤27* *w*		
				PDA:LPA ≥0.5	92	55
				PDA diameter ≥1.5 mm	75	78
				La:Ao ≥1.4	89	72
				E/A ratio >1	10	6
Smith et al.^38^	GA < 32 weeks	Echo within 48 h	PDA ≥ 2 mm on echocardiography at 1 month of age	PDA diameter >2.1 mm	78	88
				Systolic flow velocity ≤131 m/s	78	75
				Diastolic flow velocity ≤75 m/s	89	83
				Ratio systolic to diastolic flow velocity >1.9	88	90
Polat et al.^49^	GA ≤ 28 weeks BW < 1000 g	Echo within 6–12 h	PDA 72 h defined as diameter >1.5 mm and LA:Ao >1.5 OR DADF retrograde/absent OR Pulsatile flow PDA	Ductal length <5.2 mm	82	83

*Ao* aorta, *BW* birth weight, *CI Ao* cardiac index across aortic valve, *DADF* descending aorta diastolic flow, *E/A* *ratio* mitral valve early filling to atrial contraction ratio, *fE* early filling peak velocity, *GA* gestational age, *HsPDA* hemodynamic significant PDA, *LA* left atrium, *La:Ao* left atrium to aortic root ratio, *LPA* left pulmonary artery, *LVO* left ventricular output, *PDA* patent ductus arteriosus

### Guidance of treatment

The ultimate decision to treat the PDA lies in the hands of the clinician and should be based on integration of the hemodynamic indices of shunt volume within the clinical context. The approach to treatment may include shunt modulation strategies, pharmacological treatment, or surgical intervention. When pharmacological treatment is initiated, an echocardiography-guided approach has a potential to limit the number of drug doses.^63,64^

### Follow-up after treatment

It is important to highlight the role of NPE after treatment. A follow-up study after PDA treatment can be used to evaluate the presence of a pulmonary branch stenosis and confirm presence or absence of coarctation of the aorta when it is suspected in presence of a PDA. In the light of increasing popularity of a more conservative approach, a strict follow-up of the open PDA even after discharge is warranted for the detection of possible congestive heart failure.^2,65,66^

### Post-operative management

Post-ligation cardiac syndrome is a well-known entity of cardiorespiratory instability associated with surgical treatment for the PDA. It is related to a sudden change in loading conditions of the heart. Novel deformation imaging techniques have contributed considerably to the understanding of the underlying mechanism.^67^ NPE can guide the clinician in identifying patients at risk of post-operative instability. An LVO <200 mls/kg/min at 1 h post ligation predicts low cardiac output and subsequent early inotropic treatment with milrinone was associated with improved outcome.^68^ Infants with prolonged IVRT >30 ms were more likely to develop oxygenation or ventilation failure even in the presence of milrinone prophylaxis.^69^

## Advances in echocardiogram imaging

The emerging quantitative measures to characterize ventricular function and pulmonary hemodynamics may permit a more comprehensive assessment of PDA characteristics, pulmonary overcirculation, and systemic hypoperfusion, that could not be previously obtained with conventional imaging.^70^ In the early transitional period, there is a negative correlation between echocardiography-derived estimates of systemic vascular resistance and LV and septal strain values, and a positive correlation between increasing preload associated with a PDA and LV strain.^3,71,72^ The increase in preload is accompanied by an increase in strain but not strain rate, further supporting the relative lack of load dependency of strain rate imaging illustrated in animal models.^3,73^ The presence of an HsPDA also increases diastolic TDI velocities as extreme preterm infants with evidence of an HsPDA on day 5–7 had higher LV e′ (4.7 vs. 4.0 cm/s) and Septal e′ (3.9 vs. 3.3 cm/s), and a higher LV E/e′ (13 vs. 10) (all *p* < 0.05), when compared to uncomplicated preterm infants.^74^ PDA ligation has a negative impact on LV strain in the immediate post-operative period, followed by recovery 24 h later.^75^ This reduction in LV global longitudinal strain post-operatively can be attributed to the increase in afterload and the decrease in preload associated with the procedure. The increasing size of the PDA in the first 3 days of age has been correlated with increased RV areas, but stable RV fractional area of change (FAC). The RV initially pumps against the high vascular resistance in the lungs, potentially resulting in temporary morphologic changes to its structure with preserved function.^5,76^ However, a persistent HsPDA by day 5–7 will result in a lower RV FAC compared to those without a PDA (42%^7^ vs. 49%,^9^ *p* < 0.001), further highlighting the utility of RV FAC as an addition to the hemodynamic assessment of preterm infants during the first week of age.^77^

## Conclusion

Despite widespread uncertainty about the management of the PDA, NPE is increasingly recognized as an important tool for screening, diagnosing, and assessing hemodynamic significance of the PDA. The information can be used to follow-up patients and/or to decide on treatment. Although echocardiographic measurements are limited by clinical validation and most variables lack good repeatability, training frameworks, practice guidelines, and expert agreement on standardization of different variables might help to overcome these issues. Standardization of assessment would limit over-treatment and help in developing consensus approach through objective patient selection and test interventions.

## Appendix

**European Special Interest Group “Neonatologist-Performed Echocardiography” (NPE), endorsed by the European Society for Paediatric Research (ESPR) and the European Board of Neonatology (EBN)**

de Boode WP (chairman), Department of Neonatology, Radboud University Medical Center, Radboud Institute for Health Sciences, Amalia Children’s Hospital, Nijmegen, The Netherlands (willem.deboode@radboudumc.nl);

Austin T, Department of Neonatology, Rosie Hospital, Cambridge University Hospitals NHS Foundation Trust, Cambridge, UK (topun.austin@addenbrookes.nhs.uk)

Bohlin K, Department of Neonatology, Karolinska University Hospital, Karolinska Institutet, Stockholm, Sweden (kajsa.bohlin@ki.se)

Bravo MC, Department of Neonatology, La Paz University Hospital, Madrid, Spain (mcarmen.bravo@salud.madrid.org)

Breatnach CR, Department of Neonatology, The Rotunda Hospital, Dublin, Ireland (colm.breatnach@gmail.com)

Breindahl M, Karolinska University Hospital, Karolinska Institutet, Stockholm, Sweden (morten.breindahl@sll.se)

Dempsey E, INFANT Centre, Cork University Maternity Hospital, University College Cork, Ireland (g.dempsey@ucc.ie)

El-Khuffash A, Department of Neonatology, The Rotunda Hospital, Dublin, Ireland; Department of Pediatrics, The Royal College of Surgeons in Ireland, Dublin, Ireland (afifelkhuffash@rcsi.ie)

Groves AM, Division of Newborn Medicine, Mount Sinai Kravis Children’s Hospital, New York, NY, USA. (alan.groves@mssm.edu)

Gupta S, University Hospital of North Tees, Durham University, Stockton-on-Tees, UK (samir.gupta@nth.nhs.uk)

Horsberg Eriksen B, Department of Pediatrics, Møre and Romsdal Hospital Trust, Ålesund, Norway (beate.eriksen@me.com)

Levy PT, Department of Pediatrics, Washington University School of Medicine, Saint Louis, MO, USA; Department of Pediatrics, Goryeb Children’s Hospital, Morristown, NJ, USA (Levy_P@kids.wustl.edu)

McNamara PJ, Departments of Pediatrics and Physiology, University of Toronto, Toronto, ON, Canada (patrick.mcnamara@sickkids.ca)

Molnar Z, John Radcliffe Hospital, Oxford, UK (zoltan.Molnar@ouh.nhs.uk)

Nestaas E, Institute of Clinical Medicine, Faculty of Medicine, University of Oslo, Oslo, Norway; Department of Cardiology and Center for Cardiological Innovation, Oslo University Hospital, Rikshospitalet, Oslo, Norway; Department of Paediatrics, Vestfold Hospital Trust, Tønsberg, Norway (nestaas@hotmail.com)

Rogerson SR, The Royal Women’s Hospital, Parkville, VIC, Australia (sheryle.Rogerson@thewomens.org.au)

Roehr CC, Department of Paediatrics, University of Oxford, John Radcliffe Hospital, Oxford, UK (charles.roehr@paediatrics.ox.ac.uk)

Savoia M, Azienda Ospedaliero-Universitaria S. Maria della Misericordia, Udine, Italy (marilena.savoia@gmail.com)

Schubert U, Department of Clinical Science, Intervention and Technology, Karolinska Institutet, Stockholm, Sweden (ulfschubert@gmx.de)

Schwarz CE, Department of Neonatology, University Children’s Hospital of Tübingen, Tübingen, Germany (c.schwarz@med.uni-tuebingen.de)

Sehgal A, Department of Pediatrics, Monash University, Melbourne, VIC, Australia (arvind.sehgal@monash.edu)

Singh Y, Addenbrooke’s Hospital, Cambridge University Hospitals NHS Foundation Trust, Cambridge, UK (yogen.Singh@nhs.net)

Slieker MG, Department of Paediatric Cardiology, Radboudumc Amalia Children’s Hospital, Nijmegen, The Netherlands (Martijn.Slieker@radboudumc.nl)

Tissot C, Department of Pediatrics, Clinique des Grangettes, Chêne Bougeries, Switzerland (cecile.tissot@hotmail.com)

van der Lee R, Department of Neonatology, Radboud University Medical Center, Radboud Institute for Health Sciences, Amalia Children’s Hospital, Nijmegen, The Netherlands (Robin.vanderLee@radboudumc.nl)

van Laere D, Department of Pediatrics, Antwerp University Hospital UZA, Edegem, Belgium (david.VanLaere@uza.be)

van Overmeire B, Department of Neonatology, University Hospital Brussels, Brussels, Belgium (bart.van.overmeire@erasme.ulb.ac.be)

van Wyk, L.Department of Paediatrics and Child Health, University of Stellenbosch, Cape Town, South Africa (lizelle@sun.ac.za)
